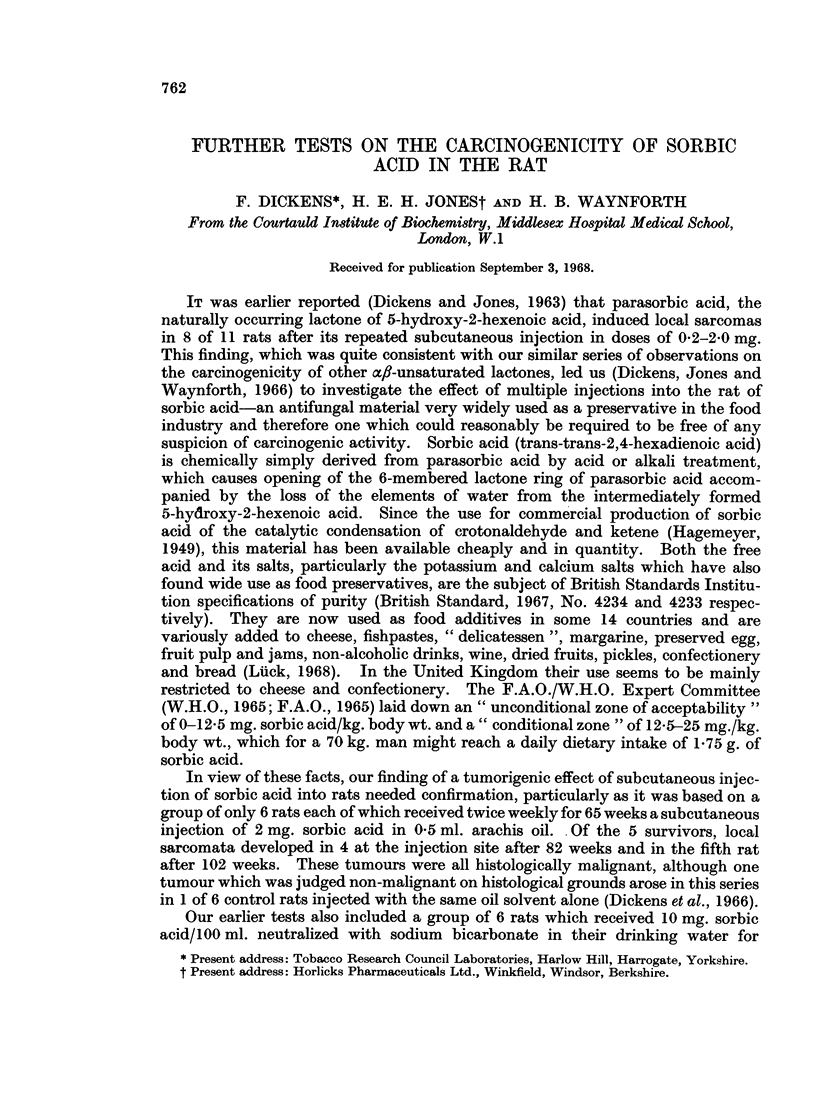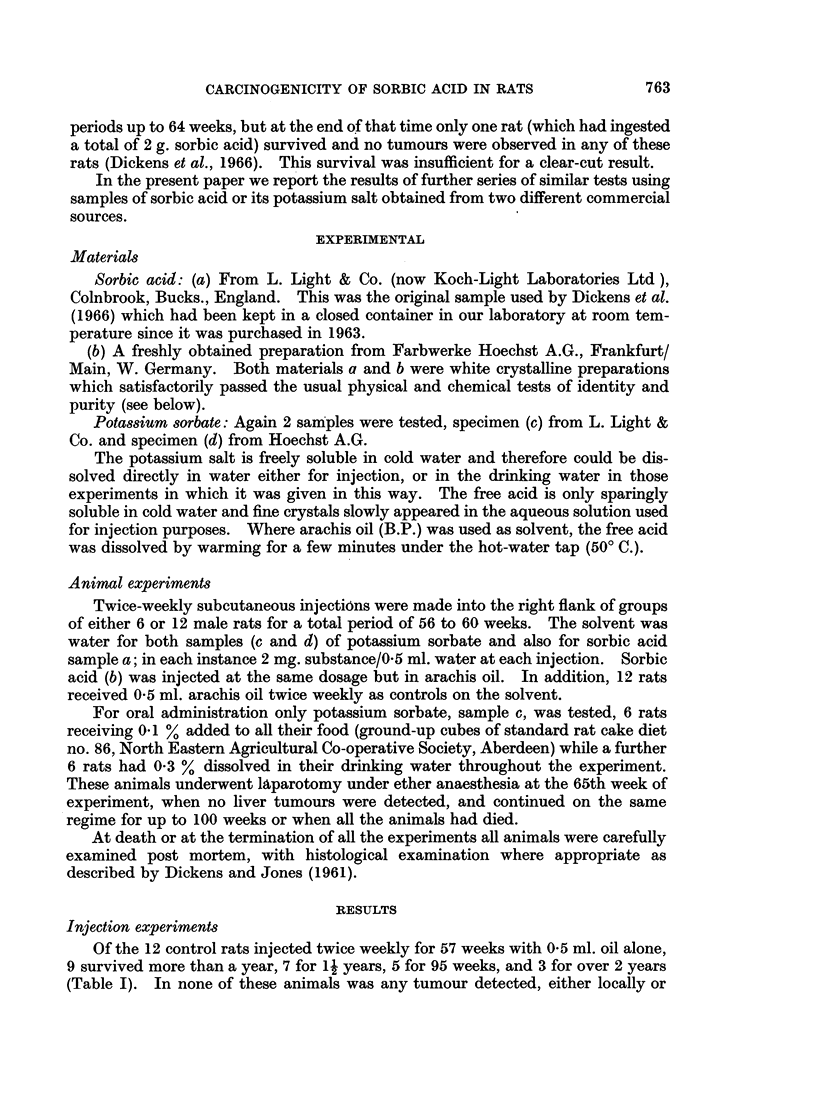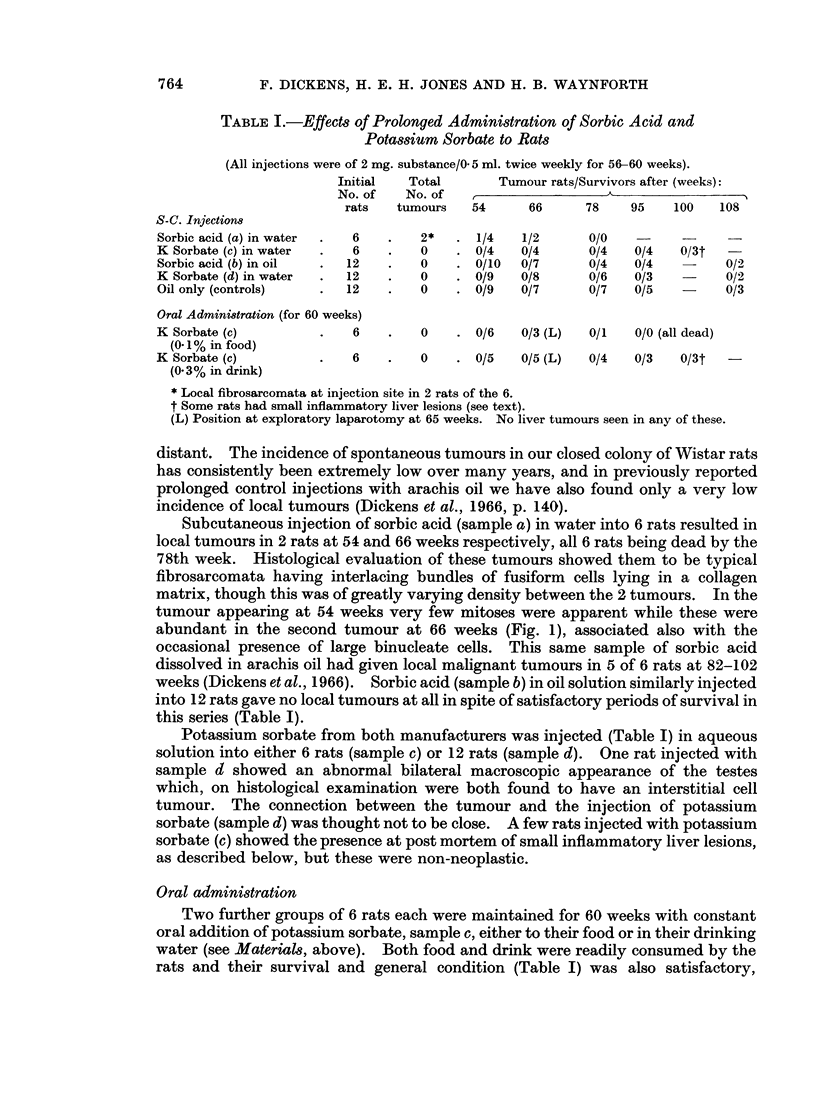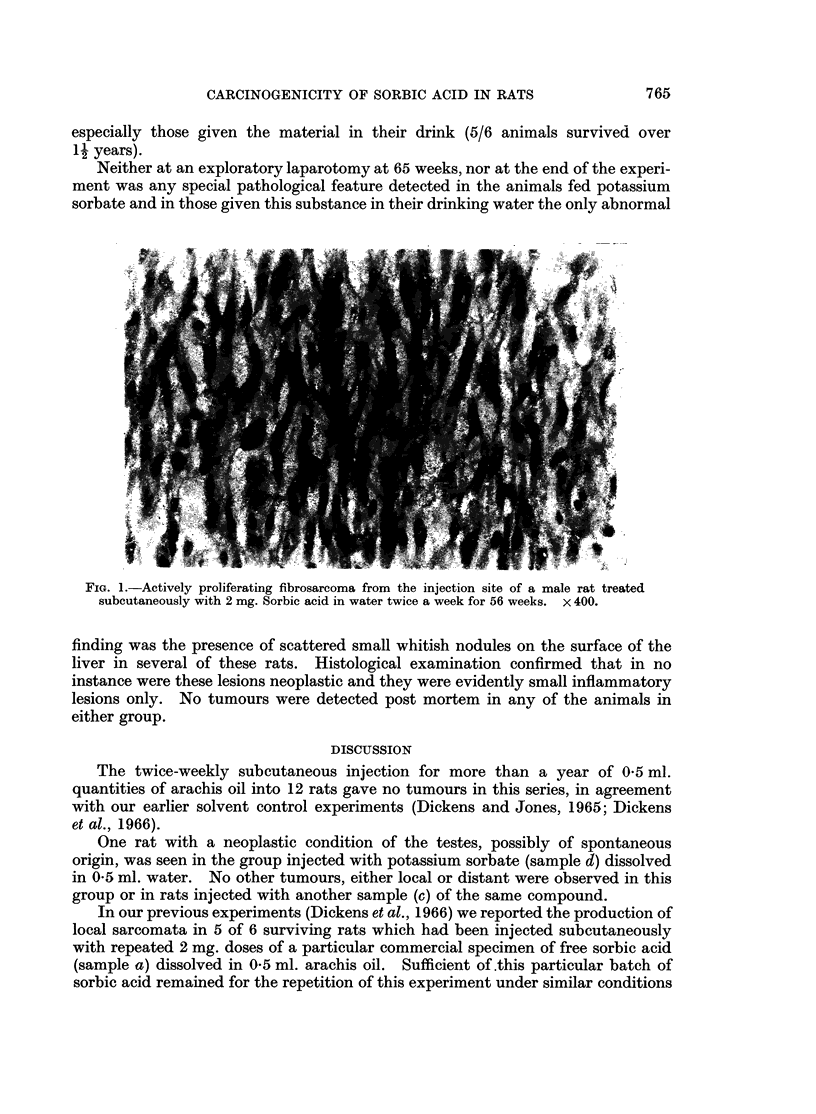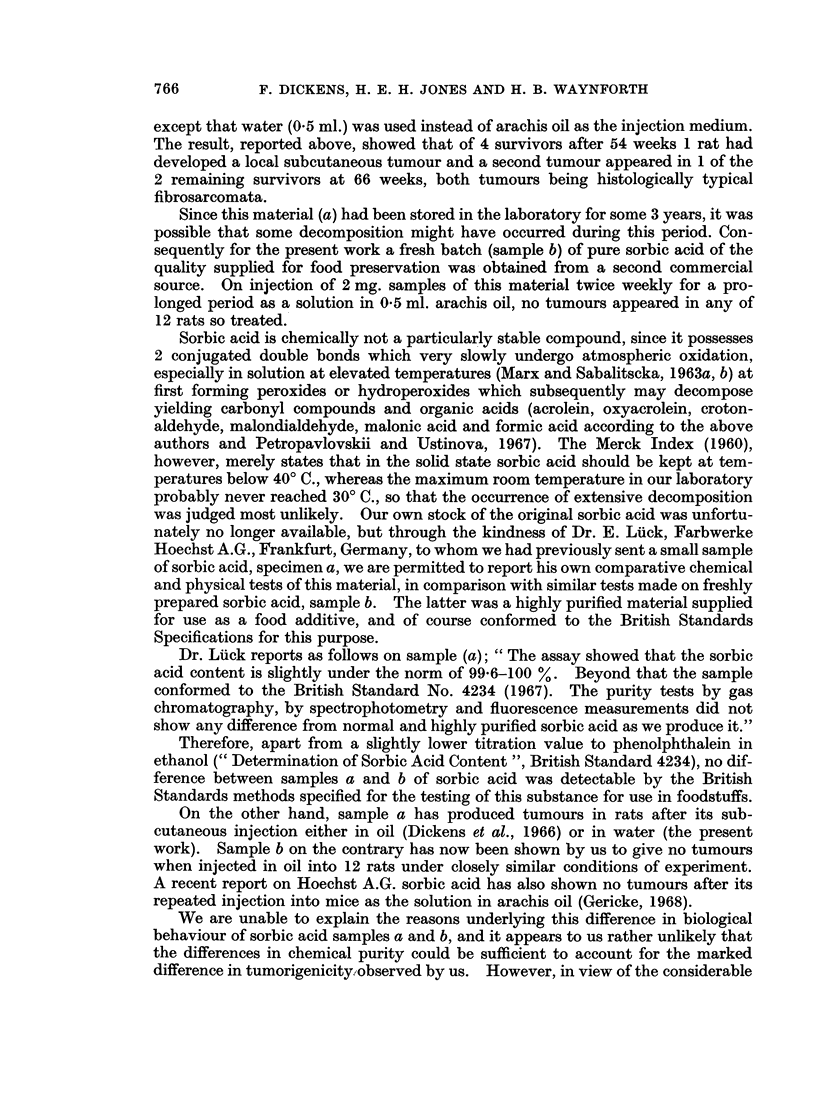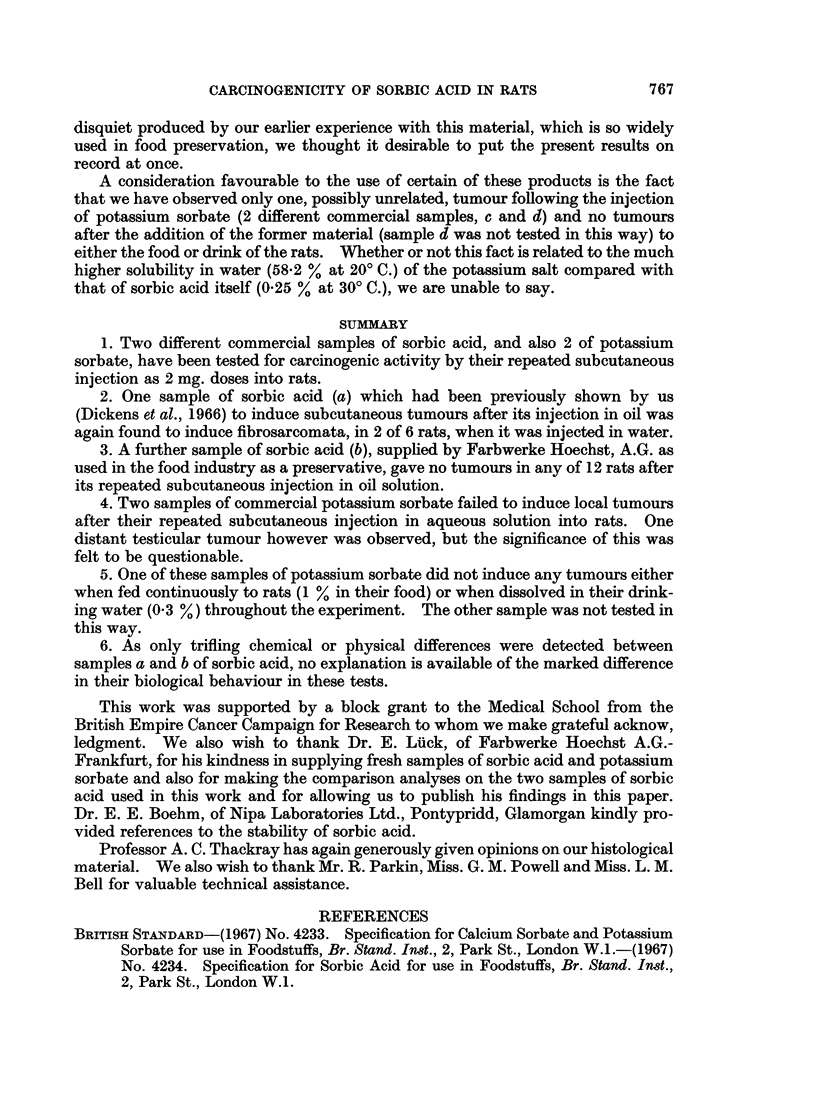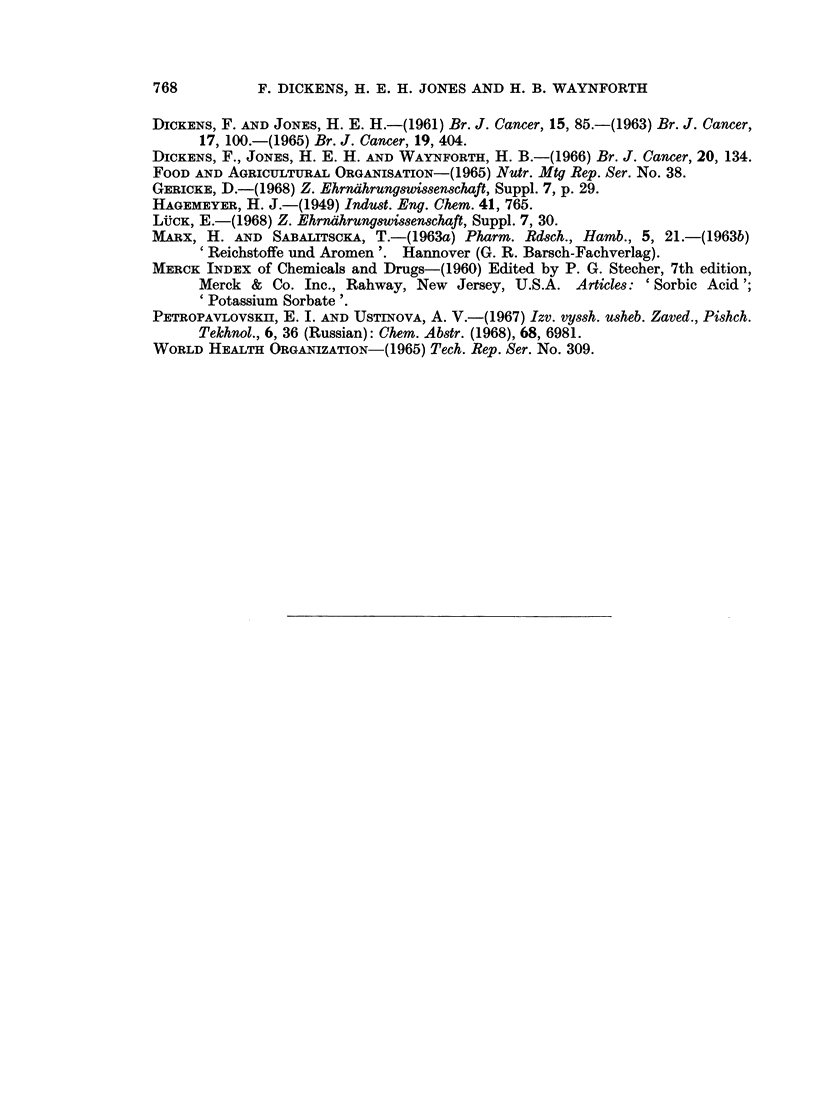# Further tests on the carcinogenicity of sorbic acid in the rat.

**DOI:** 10.1038/bjc.1968.90

**Published:** 1968-12

**Authors:** F. Dickens, H. E. Jones, H. B. Waynforth

## Abstract

**Images:**


### FURTHER TESTS ON THE CARCINOGENICITY OF SORBIC ACID IN THE RAT

F. DICKENS*, H. E. H. JONES† AND H. B. WAYNFORTH

*From the Courtauld Institute of Biochemistry, Middlesex Hospital Medical School, London, W.1*

Received for publication September 3, 1968.

IT was earlier reported (Dickens and Jones, 1963) that parasorbic acid, the naturally occurring lactone of 5-hydroxy-2-hexenoic acid, induced local sarcomas in 8 of 11 rats after its repeated subcutaneous injection in doses of 0·2–2·0 mg. This finding, which was quite consistent with our similar series of observations on the carcinogenicity of other $\alpha\beta$-unsaturated lactones, led us (Dickens, Jones and Waynforth, 1966) to investigate the effect of multiple injections into the rat of sorbic acid—an antifungal material very widely used as a preservative in the food industry and therefore one which could reasonably be required to be free of any suspicion of carcinogenic activity. Sorbic acid (trans-trans-2,4-hexadienoic acid) is chemically simply derived from parasorbic acid by acid or alkali treatment, which causes opening of the 6-membered lactone ring of parasorbic acid accompanied by the loss of the elements of water from the intermediately formed 5-hydroxy-2-hexenoic acid. Since the use for commercial production of sorbic acid of the catalytic condensation of crotonaldehyde and ketene (Hagemeyer, 1949), this material has been available cheaply and in quantity. Both the free acid and its salts, particularly the potassium and calcium salts which have also found wide use as food preservatives, are the subject of British Standards Institution specifications of purity (British Standard, 1967, No. 4234 and 4233 respectively). They are now used as food additives in some 14 countries and are variously added to cheese, fishpastes, " delicatessen ", margarine, preserved egg, fruit pulp and jams, non-alcoholic drinks, wine, dried fruits, pickles, confectionery and bread (Lück, 1968). In the United Kingdom their use seems to be mainly restricted to cheese and confectionery. The F.A.O./W.H.O. Expert Committee (W.H.O., 1965; F.A.O., 1965) laid down an " unconditional zone of acceptability " of 0–12·5 mg. sorbic acid/kg. body wt. and a " conditional zone " of 12·5–25 mg./kg. body wt., which for a 70 kg. man might reach a daily dietary intake of 1·75 g. of sorbic acid.

In view of these facts, our finding of a tumorigenic effect of subcutaneous injection of sorbic acid into rats needed confirmation, particularly as it was based on a group of only 6 rats each of which received twice weekly for 65 weeks a subcutaneous injection of 2 mg. sorbic acid in 0·5 ml. arachis oil. Of the 5 survivors, local sarcomata developed in 4 at the injection site after 82 weeks and in the fifth rat after 102 weeks. These tumours were all histologically malignant, although one tumour which was judged non-malignant on histological grounds arose in this series in 1 of 6 control rats injected with the same oil solvent alone (Dickens *et al.*, 1966).

Our earlier tests also included a group of 6 rats which received 10 mg. sorbic acid/100 ml. neutralized with sodium bicarbonate in their drinking water for

* Present address: Tobacco Research Council Laboratories, Harlow Hill, Harrogate, Yorkshire.
† Present address: Horlicks Pharmaceuticals Ltd., Winkfield, Windsor, Berkshire.

periods up to 64 weeks, but at the end of that time only one rat (which had ingested a total of 2 g. sorbic acid) survived and no tumours were observed in any of these rats (Dickens *et al.*, 1966). This survival was insufficient for a clear-cut result.

In the present paper we report the results of further series of similar tests using samples of sorbic acid or its potassium salt obtained from two different commercial sources.

### EXPERIMENTAL

### *Materials*

*Sorbic acid:* (*a*) From L. Light & Co. (now Koch-Light Laboratories Ltd ), Colnbrook, Bucks., England. This was the original sample used by Dickens *et al.* (1966) which had been kept in a closed container in our laboratory at room temperature since it was purchased in 1963.

(*b*) A freshly obtained preparation from Farbwerke Hoechst A.G., Frankfurt/ Main, W. Germany. Both materials *a* and *b* were white crystalline preparations which satisfactorily passed the usual physical and chemical tests of identity and purity (see below).

*Potassium sorbate:* Again 2 samples were tested, specimen (*c*) from L. Light & Co. and specimen (*d*) from Hoechst A.G.

The potassium salt is freely soluble in cold water and therefore could be dissolved directly in water either for injection, or in the drinking water in those experiments in which it was given in this way. The free acid is only sparingly soluble in cold water and fine crystals slowly appeared in the aqueous solution used for injection purposes. Where arachis oil (B.P.) was used as solvent, the free acid was dissolved by warming for a few minutes under the hot-water tap (50° C.).

### *Animal experiments*

Twice-weekly subcutaneous injections were made into the right flank of groups of either 6 or 12 male rats for a total period of 56 to 60 weeks. The solvent was water for both samples (*c* and *d*) of potassium sorbate and also for sorbic acid sample *a*; in each instance 2 mg. substance/0·5 ml. water at each injection. Sorbic acid (*b*) was injected at the same dosage but in arachis oil. In addition, 12 rats received 0·5 ml. arachis oil twice weekly as controls on the solvent.

For oral administration only potassium sorbate, sample *c*, was tested, 6 rats receiving 0·1 % added to all their food (ground-up cubes of standard rat cake diet no. 86, North Eastern Agricultural Co-operative Society, Aberdeen) while a further 6 rats had 0·3 % dissolved in their drinking water throughout the experiment. These animals underwent laparotomy under ether anaesthesia at the 65th week of experiment, when no liver tumours were detected, and continued on the same regime for up to 100 weeks or when all the animals had died.

At death or at the termination of all the experiments all animals were carefully examined post mortem, with histological examination where appropriate as described by Dickens and Jones (1961).

### RESULTS

### *Injection experiments*

Of the 12 control rats injected twice weekly for 57 weeks with 0·5 ml. oil alone, 9 survived more than a year, 7 for $1\frac{1}{2}$ years, 5 for 95 weeks, and 3 for over 2 years (Table I). In none of these animals was any tumour detected, either locally or

TABLE I.—*Effects of Prolonged Administration of Sorbic Acid and Potassium Sorbate to Rats*

(All injections were of 2 mg. substance/0·5 ml. twice weekly for 56–60 weeks).

| | Initial No. of rats | Total No. of tumours | Tumour rats/Survivors after (weeks): 54 | 66 | 78 | 95 | 100 | 108 |
|---|---|---|---|---|---|---|---|---|
| *S-C. Injections* | | | | | | | | |
| Sorbic acid (*a*) in water | 6 | 2* | 1/4 | 1/2 | 0/0 | — | — | — |
| K Sorbate (*c*) in water | 6 | 0 | 0/4 | 0/4 | 0/4 | 0/4 | 0/3† | — |
| Sorbic acid (*b*) in oil | 12 | 0 | 0/10 | 0/7 | 0/4 | 0/4 | — | 0/2 |
| K Sorbate (*d*) in water | 12 | 0 | 0/9 | 0/8 | 0/6 | 0/3 | — | 0/2 |
| Oil only (controls) | 12 | 0 | 0/9 | 0/7 | 0/7 | 0/5 | — | 0/3 |
| *Oral Administration* (for 60 weeks) | | | | | | | | |
| K Sorbate (*c*) (0·1% in food) | 6 | 0 | 0/6 | 0/3 (L) | 0/1 | 0/0 (all dead) | | |
| K Sorbate (*c*) (0·3% in drink) | 6 | 0 | 0/5 | 0/5 (L) | 0/4 | 0/3 | 0/3† | — |

* Local fibrosarcomata at injection site in 2 rats of the 6.
† Some rats had small inflammatory liver lesions (see text).
(L) Position at exploratory laparotomy at 65 weeks. No liver tumours seen in any of these.

distant. The incidence of spontaneous tumours in our closed colony of Wistar rats has consistently been extremely low over many years, and in previously reported prolonged control injections with arachis oil we have also found only a very low incidence of local tumours (Dickens *et al.*, 1966, p. 140).

Subcutaneous injection of sorbic acid (sample *a*) in water into 6 rats resulted in local tumours in 2 rats at 54 and 66 weeks respectively, all 6 rats being dead by the 78th week. Histological evaluation of these tumours showed them to be typical fibrosarcomata having interlacing bundles of fusiform cells lying in a collagen matrix, though this was of greatly varying density between the 2 tumours. In the tumour appearing at 54 weeks very few mitoses were apparent while these were abundant in the second tumour at 66 weeks (Fig. 1), associated also with the occasional presence of large binucleate cells. This same sample of sorbic acid dissolved in arachis oil had given local malignant tumours in 5 of 6 rats at 82–102 weeks (Dickens *et al.*, 1966). Sorbic acid (sample *b*) in oil solution similarly injected into 12 rats gave no local tumours at all in spite of satisfactory periods of survival in this series (Table I).

Potassium sorbate from both manufacturers was injected (Table I) in aqueous solution into either 6 rats (sample *c*) or 12 rats (sample *d*). One rat injected with sample *d* showed an abnormal bilateral macroscopic appearance of the testes which, on histological examination were both found to have an interstitial cell tumour. The connection between the tumour and the injection of potassium sorbate (sample *d*) was thought not to be close. A few rats injected with potassium sorbate (*c*) showed the presence at post mortem of small inflammatory liver lesions, as described below, but these were non-neoplastic.

*Oral administration*

Two further groups of 6 rats each were maintained for 60 weeks with constant oral addition of potassium sorbate, sample *c*, either to their food or in their drinking water (see *Materials*, above). Both food and drink were readily consumed by the rats and their survival and general condition (Table I) was also satisfactory, especially those given the material in their drink (5/6 animals survived over $1\frac{1}{2}$ years).

Neither at an exploratory laparotomy at 65 weeks, nor at the end of the experiment was any special pathological feature detected in the animals fed potassium sorbate and in those given this substance in their drinking water the only abnormal

FIG. 1.—Actively proliferating fibrosarcoma from the injection site of a male rat treated subcutaneously with 2 mg. Sorbic acid in water twice a week for 56 weeks. ×400.

finding was the presence of scattered small whitish nodules on the surface of the liver in several of these rats. Histological examination confirmed that in no instance were these lesions neoplastic and they were evidently small inflammatory lesions only. No tumours were detected post mortem in any of the animals in either group.

### DISCUSSION

The twice-weekly subcutaneous injection for more than a year of 0·5 ml. quantities of arachis oil into 12 rats gave no tumours in this series, in agreement with our earlier solvent control experiments (Dickens and Jones, 1965; Dickens *et al.*, 1966).

One rat with a neoplastic condition of the testes, possibly of spontaneous origin, was seen in the group injected with potassium sorbate (sample *d*) dissolved in 0·5 ml. water. No other tumours, either local or distant were observed in this group or in rats injected with another sample (*c*) of the same compound.

In our previous experiments (Dickens *et al.*, 1966) we reported the production of local sarcomata in 5 of 6 surviving rats which had been injected subcutaneously with repeated 2 mg. doses of a particular commercial specimen of free sorbic acid (sample *a*) dissolved in 0·5 ml. arachis oil. Sufficient of this particular batch of sorbic acid remained for the repetition of this experiment under similar conditions except that water (0·5 ml.) was used instead of arachis oil as the injection medium. The result, reported above, showed that of 4 survivors after 54 weeks 1 rat had developed a local subcutaneous tumour and a second tumour appeared in 1 of the 2 remaining survivors at 66 weeks, both tumours being histologically typical fibrosarcomata.

Since this material (*a*) had been stored in the laboratory for some 3 years, it was possible that some decomposition might have occurred during this period. Consequently for the present work a fresh batch (sample *b*) of pure sorbic acid of the quality supplied for food preservation was obtained from a second commercial source. On injection of 2 mg. samples of this material twice weekly for a prolonged period as a solution in 0·5 ml. arachis oil, no tumours appeared in any of 12 rats so treated.

Sorbic acid is chemically not a particularly stable compound, since it possesses 2 conjugated double bonds which very slowly undergo atmospheric oxidation, especially in solution at elevated temperatures (Marx and Sabalitscka, 1963*a*, *b*) at first forming peroxides or hydroperoxides which subsequently may decompose yielding carbonyl compounds and organic acids (acrolein, oxyacrolein, crotonaldehyde, malondialdehyde, malonic acid and formic acid according to the above authors and Petropavlovskii and Ustinova, 1967). The Merck Index (1960), however, merely states that in the solid state sorbic acid should be kept at temperatures below 40° C., whereas the maximum room temperature in our laboratory probably never reached 30° C., so that the occurrence of extensive decomposition was judged most unlikely. Our own stock of the original sorbic acid was unfortunately no longer available, but through the kindness of Dr. E. Lück, Farbwerke Hoechst A.G., Frankfurt, Germany, to whom we had previously sent a small sample of sorbic acid, specimen *a*, we are permitted to report his own comparative chemical and physical tests of this material, in comparison with similar tests made on freshly prepared sorbic acid, sample *b*. The latter was a highly purified material supplied for use as a food additive, and of course conformed to the British Standards Specifications for this purpose.

Dr. Lück reports as follows on sample (*a*); " The assay showed that the sorbic acid content is slightly under the norm of 99·6–100 %. Beyond that the sample conformed to the British Standard No. 4234 (1967). The purity tests by gas chromatography, by spectrophotometry and fluorescence measurements did not show any difference from normal and highly purified sorbic acid as we produce it."

Therefore, apart from a slightly lower titration value to phenolphthalein in ethanol (" Determination of Sorbic Acid Content ", British Standard 4234), no difference between samples *a* and *b* of sorbic acid was detectable by the British Standards methods specified for the testing of this substance for use in foodstuffs.

On the other hand, sample *a* has produced tumours in rats after its subcutaneous injection either in oil (Dickens *et al.*, 1966) or in water (the present work). Sample *b* on the contrary has now been shown by us to give no tumours when injected in oil into 12 rats under closely similar conditions of experiment. A recent report on Hoechst A.G. sorbic acid has also shown no tumours after its repeated injection into mice as the solution in arachis oil (Gericke, 1968).

We are unable to explain the reasons underlying this difference in biological behaviour of sorbic acid samples *a* and *b*, and it appears to us rather unlikely that the differences in chemical purity could be sufficient to account for the marked difference in tumorigenicity observed by us. However, in view of the considerable disquiet produced by our earlier experience with this material, which is so widely used in food preservation, we thought it desirable to put the present results on record at once.

A consideration favourable to the use of certain of these products is the fact that we have observed only one, possibly unrelated, tumour following the injection of potassium sorbate (2 different commercial samples, *c* and *d*) and no tumours after the addition of the former material (sample *d* was not tested in this way) to either the food or drink of the rats. Whether or not this fact is related to the much higher solubility in water (58·2 % at 20° C.) of the potassium salt compared with that of sorbic acid itself (0·25 % at 30° C.), we are unable to say.

SUMMARY

1. Two different commercial samples of sorbic acid, and also 2 of potassium sorbate, have been tested for carcinogenic activity by their repeated subcutaneous injection as 2 mg. doses into rats.

2. One sample of sorbic acid (*a*) which had been previously shown by us (Dickens *et al.*, 1966) to induce subcutaneous tumours after its injection in oil was again found to induce fibrosarcomata, in 2 of 6 rats, when it was injected in water.

3. A further sample of sorbic acid (*b*), supplied by Farbwerke Hoechst, A.G. as used in the food industry as a preservative, gave no tumours in any of 12 rats after its repeated subcutaneous injection in oil solution.

4. Two samples of commercial potassium sorbate failed to induce local tumours after their repeated subcutaneous injection in aqueous solution into rats. One distant testicular tumour however was observed, but the significance of this was felt to be questionable.

5. One of these samples of potassium sorbate did not induce any tumours either when fed continuously to rats (1 % in their food) or when dissolved in their drinking water (0·3 %) throughout the experiment. The other sample was not tested in this way.

6. As only trifling chemical or physical differences were detected between samples *a* and *b* of sorbic acid, no explanation is available of the marked difference in their biological behaviour in these tests.

This work was supported by a block grant to the Medical School from the British Empire Cancer Campaign for Research to whom we make grateful acknow, ledgment. We also wish to thank Dr. E. Lück, of Farbwerke Hoechst A.G.-Frankfurt, for his kindness in supplying fresh samples of sorbic acid and potassium sorbate and also for making the comparison analyses on the two samples of sorbic acid used in this work and for allowing us to publish his findings in this paper. Dr. E. E. Boehm, of Nipa Laboratories Ltd., Pontypridd, Glamorgan kindly provided references to the stability of sorbic acid.

Professor A. C. Thackray has again generously given opinions on our histological material. We also wish to thank Mr. R. Parkin, Miss. G. M. Powell and Miss. L. M. Bell for valuable technical assistance.

### REFERENCES

BRITISH STANDARD—(1967) No. 4233. Specification for Calcium Sorbate and Potassium Sorbate for use in Foodstuffs, *Br. Stand. Inst.*, 2, Park St., London W.1.—(1967) No. 4234. Specification for Sorbic Acid for use in Foodstuffs, *Br. Stand. Inst.*, 2, Park St., London W.1.

DICKENS, F. AND JONES, H. E. H.—(1961) *Br. J. Cancer*, **15**, 85.—(1963) *Br. J. Cancer*, **17**, 100.—(1965) *Br. J. Cancer*, **19**, 404.
DICKENS, F., JONES, H. E. H. AND WAYNFORTH, H. B.—(1966) *Br. J. Cancer*, **20**, 134.
FOOD AND AGRICULTURAL ORGANISATION—(1965) *Nutr. Mtg Rep. Ser.* No. 38.
GERICKE, D.—(1968) *Z. Ehrnährungswissenschaft*, Suppl. **7**, p. 29.
HAGEMEYER, H. J.—(1949) *Indust. Eng. Chem.* **41**, 765.
LÜCK, E.—(1968) *Z. Ehrnährungswissenschaft*, Suppl. **7**, 30.
MARX, H. AND SABALITSCKA, T.—(1963*a*) *Pharm. Rdsch., Hamb.*, **5**, 21.—(1963*b*) ' Reichstoffe und Aromen '. Hannover (G. R. Barsch-Fachverlag).
MERCK INDEX of Chemicals and Drugs—(1960) Edited by P. G. Stecher, 7th edition, Merck & Co. Inc., Rahway, New Jersey, U.S.A. *Articles:* ' Sorbic Acid '; ' Potassium Sorbate '.
PETROPAVLOVSKII, E. I. AND USTINOVA, A. V.—(1967) *Izv. vyssh. usheb. Zaved., Pishch. Tekhnol.*, **6**, 36 (Russian): *Chem. Abstr.* (1968), **68**, 6981.
WORLD HEALTH ORGANIZATION—(1965) *Tech. Rep. Ser.* No. 309.